# Persistent Donor Cell Gene Expression among Human Induced Pluripotent Stem Cells Contributes to Differences with Human Embryonic Stem Cells

**DOI:** 10.1371/journal.pone.0008975

**Published:** 2010-02-01

**Authors:** Zhumur Ghosh, Kitchener D. Wilson, Yi Wu, Shijun Hu, Thomas Quertermous, Joseph C. Wu

**Affiliations:** 1 Department of Medicine, Stanford University School of Medicine, Stanford, California, United States of America; 2 Department of Radiology, Stanford University School of Medicine, Stanford, California, United States of America; 3 Department of Bioengineering, Stanford University School of Medicine, Stanford, California, United States of America; 4 Institute for Stem Cell Biology and Regenerative Medicine, Stanford University School of Medicine, Stanford, California, United States of America; University of Calgary, Canada

## Abstract

Human induced pluripotent stem cells (hiPSCs) generated by de-differentiation of adult somatic cells offer potential solutions for the ethical issues surrounding human embryonic stem cells (hESCs), as well as their immunologic rejection after cellular transplantation. However, although hiPSCs have been described as “embryonic stem cell-like”, these cells have a distinct gene expression pattern compared to hESCs, making incomplete reprogramming a potential pitfall. It is unclear to what degree the difference in tissue of origin may contribute to these gene expression differences. To answer these important questions, a careful transcriptional profiling analysis is necessary to investigate the exact reprogramming state of hiPSCs, as well as analysis of the impression, if any, of the tissue of origin on the resulting hiPSCs. In this study, we compare the gene profiles of hiPSCs derived from fetal fibroblasts, neonatal fibroblasts, adipose stem cells, and keratinocytes to their corresponding donor cells and hESCs. Our analysis elucidates the overall degree of reprogramming within each hiPSC line, as well as the “distance” between each hiPSC line and its donor cell. We further identify genes that have a similar mode of regulation in hiPSCs and their corresponding donor cells compared to hESCs, allowing us to specify core sets of donor genes that continue to be expressed in each hiPSC line. We report that residual gene expression of the donor cell type contributes significantly to the differences among hiPSCs and hESCs, and adds to the incompleteness in reprogramming. Specifically, our analysis reveals that fetal fibroblast-derived hiPSCs are closer to hESCs, followed by adipose, neonatal fibroblast, and keratinocyte-derived hiPSCs.

## Introduction

Human embryonic stem cells (hESCs) are widely recognized as a precious biological source of pluripotent cells, and hold tremendous therapeutic promise due to their ability to self-renew, proliferate, and differentiate [1]. However, the use of human embryos is controversial, and the problem of immune rejection following transplantation in patients remains difficult to solve. The discovery that mouse and human somatic cells can be reprogrammed into induced pluripotent stem cells (iPSCs) has given researchers a non-controversial alternative source of pluripotent human cells. Further, iPSC technology could overcome some of the obstacles associated with immune rejection after transplantation[2], [3], [4], [5].

The direct reprogramming of somatic cells to pluripotent state was accomplished in 2006, when Takahashi and Yamanaka converted adult mouse fibroblasts to iPSCs through ectopic expression of a group of transcription factors [6]. Since then, a plethora of reports have been published showing derivation of iPSCs from various murine and human tissues [6], [7], [8], [9], [10], including human iPSCs (hiPSCs) that were derived from multiple cell types [8], [9], [10], [11], [12], [13], [14], [15], [16], [17].

In the journey of reprogramming, cells start from a differentiated state to reach an embryonic-like state after over-expression of a defined set of transcription factors that act as arbiters in the journey [6]. But pressing scientific questions remain. For instance, how close are these iPSCs to their conventional hESC counterparts? What is the exact genetic status of these reprogrammed cells? Do they still bear any “footprint” of their tissue of origin that may contribute to differences with hESCs [18]? hiPSCs at different passages have significant differences in gene expression from hESCs [19], and it has been shown that there is significant variation in the teratoma forming propensities of iPSCs depending on the tissue of origin [20], [21], [22]. With these issues in mind, Maherali and Hochedlinger published a timely and valuable review that suggests basic criteria for evaluating the pluripotency of iPSCs [23]. Hence, as the potential of hiPSCs and their derivatives for regenerative medicine is being evaluated, it has become clear that an analysis is needed of the overall state of these cells, as well as comparisons with other derived lines, in order to evaluate their safety for regenerative therapy.

Although most publications report that the gene expression profiles of hiPSCs are “nearly identical” to their embryo-derived counterparts, hESCs, it is essential to clearly define the differences between them. The quantity of gene expression differences between the two cellular populations, and among the hiPSCs themselves, could account for incomplete reprogramming. Therefore, we believe that a careful analysis is necessary in order to discern whether hiPSCs bear persistent donor cell gene expression which may interfere with their reversion from somatic cells.

We performed a comprehensive transcriptional analysis of different hiPSC lines that have been previously reported to be derived from several different cell sources, using hESCs as a gold standard. The sum of our analysis has uncovered a persistent gene expression pattern in hiPSCs that appears to be related to the specific tissue of origin. Bioinformatic analysis reveals a degree of incompleteness in reprogramming that results from this residual gene expression. In the future, further investigation is warranted to determine whether persistent donor cell gene expression in hiPSCs could cause functional differences in their pluripotency and capacity to differentiate into their original cell type rather than compared to other cell types.

## Materials and Methods

### Source of Gene Profiles

In order to compute the distance between the “hiPSC-state”, “hESC-state”, and “differentiated state”, we analyzed the transcriptional profiles of previously reported hiPSC lines [11], [12], [15], [17] and compared their gene expression data to those of multiple hESCs and donor cell lines. Gene expression data were obtained from the Gene Expression Omnibus (GEO) repository (http://www.ncbi.nlm.nih.gov/geo/), which is currently the largest fully public gene expression resource. The GEO [24] repository at the National Center for Biotechnology Information (NCBI) archives and freely disseminates microarray and other forms of high-throughput data generated by the scientific community. **Table 1** summarizes the details of the hiPSC lines considered in our analysis, including the nomenclature we used for each cell line. The 6 hESC lines considered in our analysis are H1, H7, H9, H13, H14, and T3, all of which are also derived from GEO repository.

**Table 1:** Summary of the hiPSC lines used for analyzing donor cell vs. hiPSC relationship.

Donor Cells	Donor cell line nomenclature	Corresponding iPS cell line nomenclature	Reprogramming method	References
Foreskin fibroblast	hFFib	iPS-hFFib	Reprogramming by using non-integrating oriP/EBNA1-based episomal vectors	[17]
Adipose stem cells	hASC	iPS-hASC	Reprogramming by lentiviral transduction with Oct4, Sox2, Klf4, and c-MYC	[15]
Neonatal fibroblast	hNFib	iPS-hNFib	Direct delivery of defined reprogramming proteins.	[12]
Keratinocytes	hKT	iPS-hKT	Reprogramming by retroviral transduction with Oct4, Sox2, Klf4, and c-Myc	[11]

The six hESC lines used in this study are H1, H7, H9, H13, H14, and T3.

### Microarray Analysis

All gene expression data were reported to be obtained with the HG-U133plus2 microarray platform (Affymetrix). Note that the data on adipose stem cell derived-hiPSCs reported by Sun et al.'s paper [15] used the Agilent 4×44 K whole human genome microarray platform. For our study, we re- hybridized the same RNA samples to the Affymetrix HG-U133Plus2 chips, and the expression signals were scanned on an Affymetrix GeneChip Scanner. All data sets were analyzed using GeneSpring GX 10.0 software (Agilent Technologies, Inc. www.chem.agilent.com). Gene-level signal estimates were derived from the CEL files. Summarization of gene expression data was performed by implementing the robust multichip averaging algorithm, with subsequent baseline normalization of the log-summarized values for each probe set to that of the median log summarized value for the same probe set in the control group. Expression data were then filtered to remove probe sets for which the signal intensities for all the treatment groups were in the lowest 20 percentile of all intensity values. The data were then subjected to analysis of variance (ANOVA), incorporating the Benjamini–Hochberg FDR multiple testing correction, with a significance level of *P*-value <0.05 to get the differentially expressed genes between different groups. Probe sets were further filtered on the basis of a fold-change cut off of 2.0. Hierarchical clustering was performed by complete linkage [25] and uncentered correlation using the open source clustering software Cluster 3.0; results were visualized using Java TreeView [26].

### Distance Metric

We have defined the distance metric between two groups of cells to be the percentage of genes that are differentially expressed between them; thus, two “closer” groups will have a lower percentage of genes that are different between them, and *vice versa*. After gene expression data from all groups (hESC, hiPSCs and donor cells) were subjected to the same statistical screening criterion (*P*-value cut off <0.05 and a fold-change cut off of 2.0), we then calculated the distances among them. This gives a clear estimation of the status of the hiPSCs and donor cells with respect to hESCs. Furthermore, it also gives a clear idea of how closer each hiPSCs are from their corresponding donor cells compared to other donor cell types. To calculate the relative distances among hESCs, hiPSCs, and donor cells, we considered 1 to be the total proportion of genes that are significantly different between starting position (donor cells) and final position (hESC). With respect to this, we have calculated the proportion of the genes different between hiPSC and donor cells, and between hiPSCs and hESCs. This provides the status of reprogramming of hiPSCs relative to the donor cells and hESCs. Note that at present there is no uniformly accepted, epistemologically pure meaning of ‘distances’ between gene expression profiles, and different metrics are useful in different situations. Hence, it is important to choose a metric that is intuitively conceivable and has a straightforward definition, as is the case with the measure used here. However, in future studies, it will have to be compared to other distance measures addressing the same type of question.

### Functional Analysis

In order to perform functional annotation of the differentially expressed genes between different groups, we used Ingenuity Pathway Analysis (IPA) software. This software assigns biological functions to genes using the Ingenuity Pathways Knowledge Base (Ingenuity Systems, Inc., Redwood City, CA). The knowledge base comprises information about thousands of human, mouse, and rat genes [27]. This information is used to form networks to create an ‘interactome’ of genes all involved in specific biological processes.

## Results

The four different human cell sources used in our analysis are fetal fibroblasts, neonatal fibroblasts, adipose stem cells, and keratinocytes. Fibroblasts are ubiquitous terminally differentiated mesenchymal cells with multiple functions during development, tissue repair, and disease. Further, there are significant gene expression differences between fetal and neonatal fibroblasts [28]. Human adipose stem cells are a heterogeneous group of multipotent progenitor cells that are derived from adipose tissue of adult humans [29] and can differentiate into adipogenic, osteogenic, chondrogenic, and myogenic cell lineages [30]. Keratinocytes are keratin-dense epithelial cells which generate the outer protective epidermal barrier of the skin surface and appendages, a life-long process owing to the presence of self-renewing keratinocyte stem cells. These cells produce transit-amplifying cells that subsequently exit the cell cycle as they terminally differentiate [31]. Hence, it is likely that all these donor cell types possess their own distinctive epigenetic landscapes based on various DNA and histone modifications. Here we analyze the reprogramming status of the hiPSCs derived from these different types of cell.

### Defining hiPSC State Based on the Global Gene Expression Pattern

**Figure 1:**
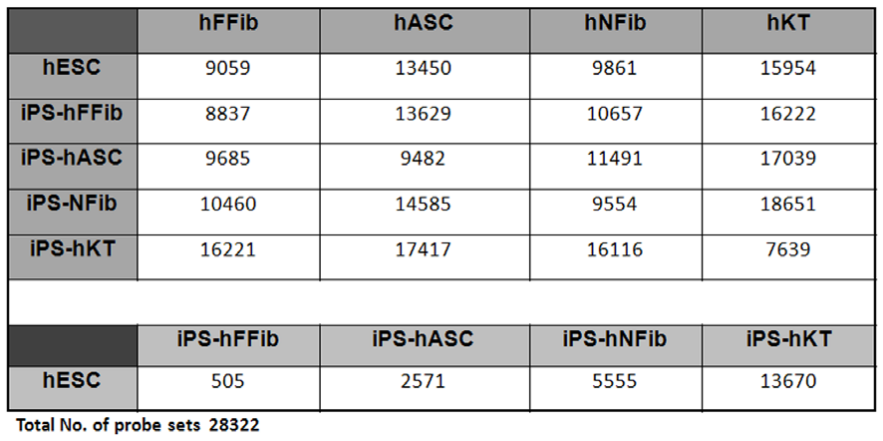
Matrix showing the number of differentially expressed genes *P*<0.05 and fold-change ≥2 across hESCs, hiPSCs, and donor cell lines.

To determine the degree of reprogramming within hiPSCs, we analyzed genome-wide expression patterns in six different hESC lines (H1, H7, H9, H13, H14, and T3), hiPSCs from fetal fibroblasts (iPS-hFFib), hiPSCs from neonatal fibroblasts (iPS-hNFib), hiPSCs from adipose stem cells (iPS-hASC), hiPSCs from keratinocytes (iPS-hKT), and their corresponding donor cells (hFFib, hNFib, hASC, and hKT). According to the distance metric defined in the methods section, a lower percentage of differentially expressed genes between two groups make them closer compared to other cell types, and *vice versa*. The matrix in **Figure 1** summarizes the probe sets retained in each groups after ANOVA analysis with cutoff of *P*-value <0.05 and fold-change of ≥2. After analyzing the expression difference between hESCs (averaged over the six different cell lines) and different hiPSC lines derived from different cell sources, we found that 505, 2571, 5555, and 13670 genes (out of 28322) were significantly different in iPS-hFFib, iPS-hASC, iPS-hNFib and iPS-hKT, respectively, compared to hESCs. Furthermore, we compared the gene expression profiles of the donor cell lines with respect to hESCs, and found that 9059, 13450, 9861, and 15954 genes (out of 28322) were significantly different in hFFib, hASC, hNFib, and hKT, respectively (**Figure 1**).

**Figure 2:**
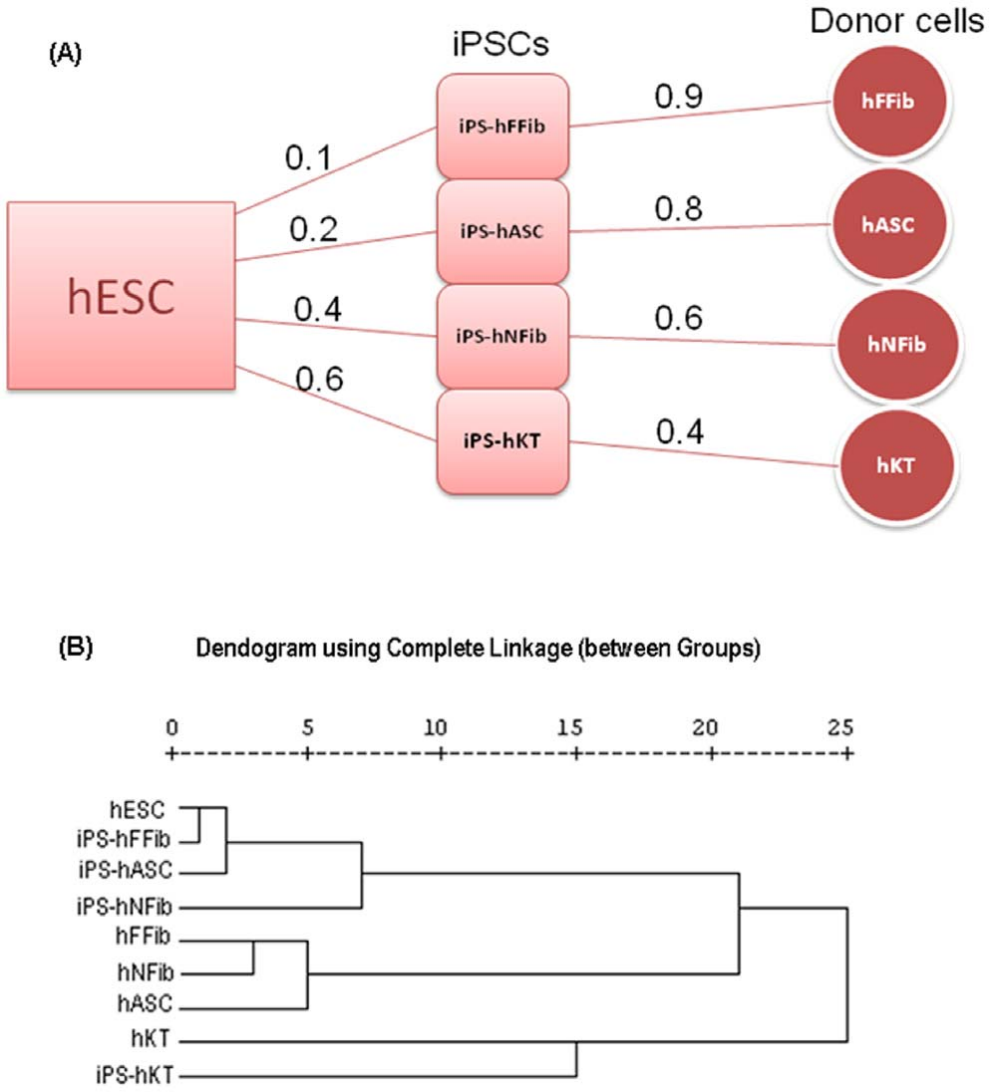
Distance between hiPSC, hESC and donor cells. (**A**) Relative distances of the hiPSC states from the corresponding somatic states (donor cells), and from the hESC state. (**B**) Global clustering among hESC, hiPSC, and donor cells.

**Figure 3:**
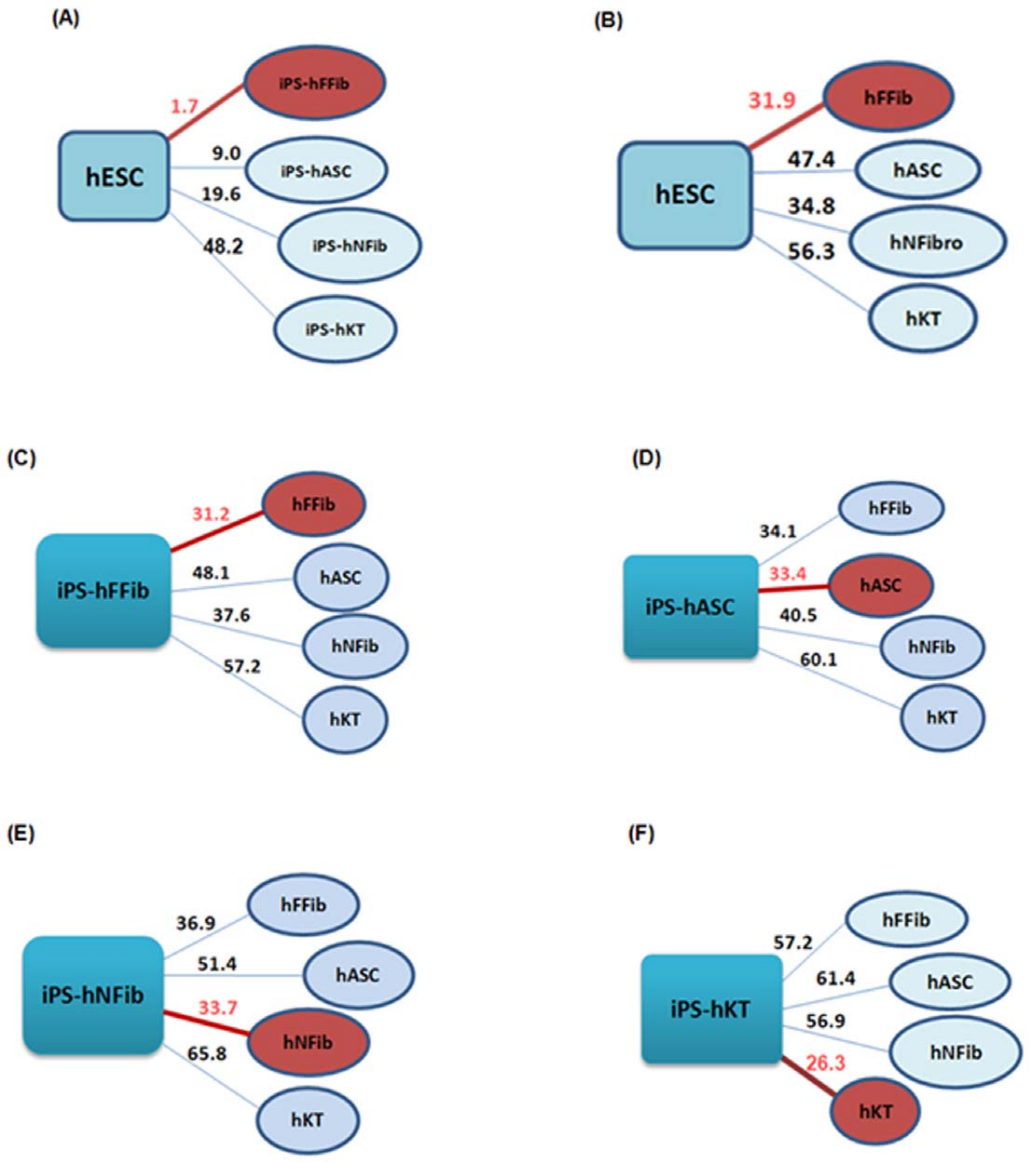
Percentage of differentially expressed genes defines the degree of dissimilarity between hESCs and hiPSCs, hESCs with the donor cell types, and hiPSCs with the 4 different donor cell types. (**A**) The distance between hESCs and hiPSCs shows iPS-hFFib to be closest to hESCs. (**B**) The distance between hESCs and donor cell types shows hFFib to be closest to hESCs. The distance between hiPSCs and 4 different donor cell types shows (**C**) iPS-hFFib closest to hFFib; (**D**) iPS-hASC closest to hASC; (**E**) iPS-hNFib closest to hNFib; and (**F**) iPS-hKT closest to hKT. (Closest grouping is marked with a red circle).

**Figure 2A** shows the relative distances between the different hiPSCs from hESCs, and from donor cells. Hierarchical clustering (**Figure 2B**) based on the global gene expression pattern, as well as distance measures (**Figures 3A and 3B** **)**, shows the differences between various hiPSCs and donor cells with respect to hESCs. These figures show that fetal fibroblast-derived hiPSCs attain a pluripotent state that is closest to hESCs, whereas keratinocyte-derived hiPSCs attain a pluripotent state that is farthest from hESCs. Furthermore, iPS-hASC is the second closest hiPSC state to hESC, followed by iPS-hNFib. Similarly, we found that different donor cells have varying distances from hESCs, as shown in **Figure 3B**.

### The Relationship between hiPSCs and Donor Cells

**Figure 4:**
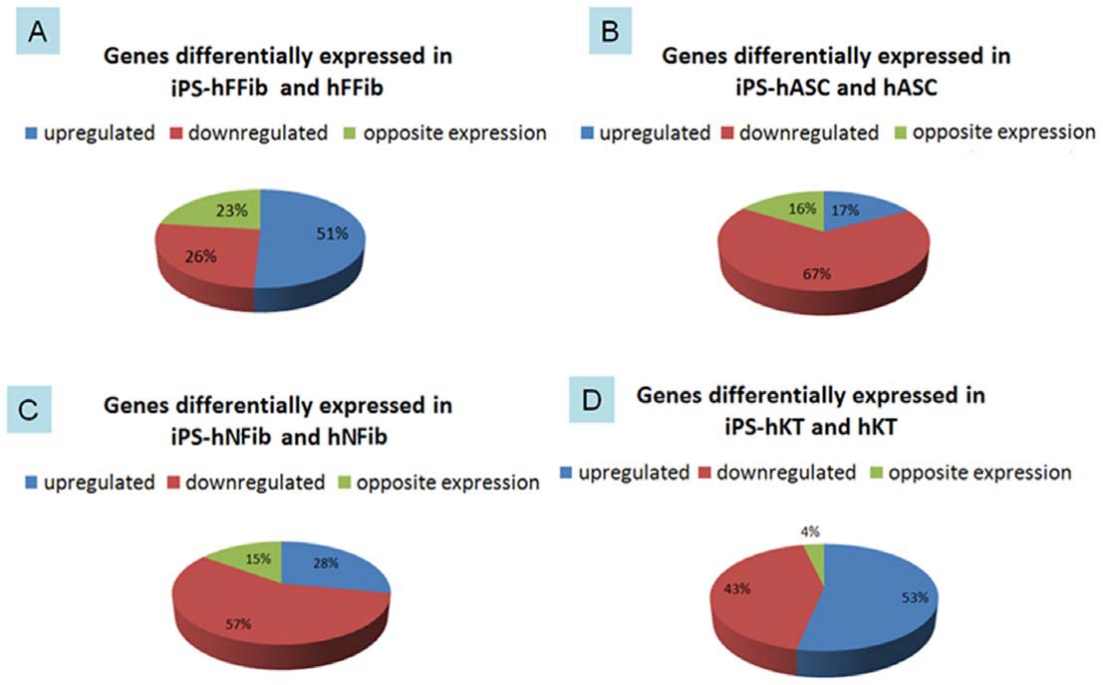
Modes of regulation of the differentially expressed genes across different hiPSCs and their corresponding donor cells compared to hESCs. 77% of the genes have similar expression pattern in iPS-hFFib and hFFib (both upregulated and downreglated). 84% of the genes have similar expression pattern in iPS-hASC and hASC. 85% of the genes have similar expression pattern in iPS-hNFib and hNFib. 96% of the genes have similar expression pattern in iPS-hKT and hKT.

To look deeper into the hiPSC and donor cell relationship, we further analyzed the genes that were *differentially* expressed between hiPSCs and their donor cells (**Figure 3C–3F**). Overall, we found that hiPSCs tend to be closer to their corresponding donor cell type than to other donor cell types. This pattern suggests that the expression of genes determining the differentiated state are not completely switched off. In order to determine the specific subset of genes that have similar modes of regulation in both hiPSCs and donor cells, we next compared the gene expression differences between hiPSCs and donor cells with respect to expression levels in hESCs. These specific gene sets hold critical clues as to how these hiPSCs retain a “memory” of their tissue of origin even after undergoing reprogramming. Within the total set of genes differentially expressed in hiPSCs and donor cells compared to hESCs, there are gene sets that are either upregulated or downregulated in both hiPSCs and donor cells. Interestingly, a third subset of genes exhibits opposing expression in between hiPSCs and donor cells. **Figure 4** depicts the distribution of the genes according to their mode of regulation in each set of hiPSC and corresponding donor cells. Among the whole set of differentially expressed genes in hiPSCs and donor cells (compared to hESCs), 77% (51% upregulated+26% downregulated), 84% (17%+67%), 85% (28%+57%), and 96% (53%+43%) of the genes have similar modes of expression in iPS-hFFib, iPS-hASC, iPS-hNFib, and iPS-hKT and their corresponding donor cells, respectively. Thus, the more completely a somatic cell is reprogrammed, the more likely its resulting hiPSC will have a distinct gene expression pattern from it. The degree of reprogramming thus determines the extent of gene expression differences between the parental and reprogrammed cells. This is clearly shown in **Figure 4**, where iPS-hFFib contains the lowest percentage of similar gene expression modes with its corresponding donor cell (77%), but iPS-hKT has the highest percentage of similar gene expression modes with its corresponding donor cell (96%). These results agree with our distance measurements between different hiPSCs and hESC, shown above (**Figure 2A and 2B**).

### Upregulated Genes in hiPSCs and Donor Cells

**Figure 5:**
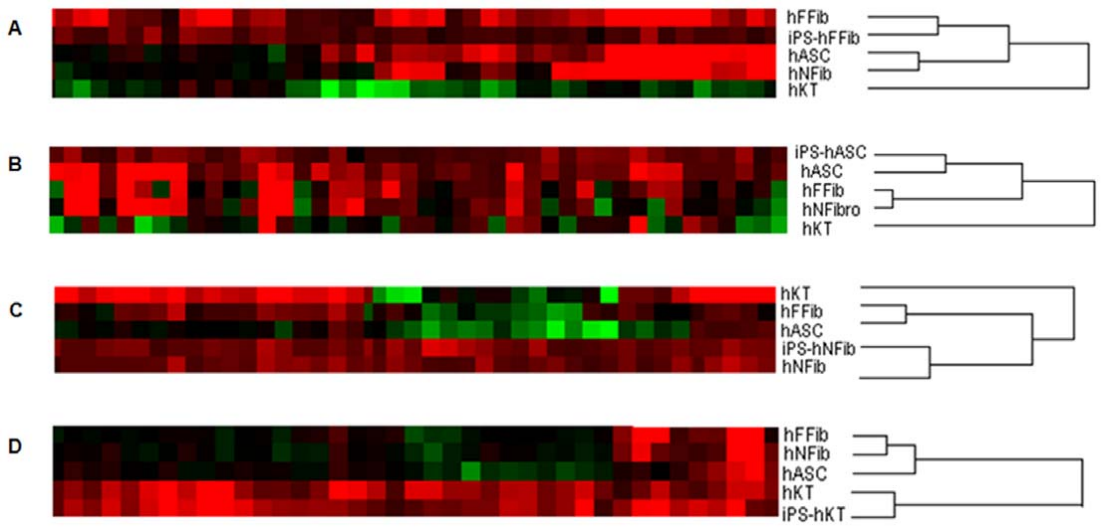
Hierarchical clustering of upregulated genes in the hiPSC and donor cells. (**A**) iPS-hFFib and hFFib. (**B**) iPS-hASC and hASC. (**C**) iPS-hNFib and hNFib. (**D**) iPS-hKT and hKT. Hierarchical clustering of the upregulated gene expression data shows that hiPSCs cluster more closely to their corresponding donor cells. This demonstrates that reprogrammed hiPSCs exhibit persistent gene expression from their corresponding donor cells.

**Figure 6:**
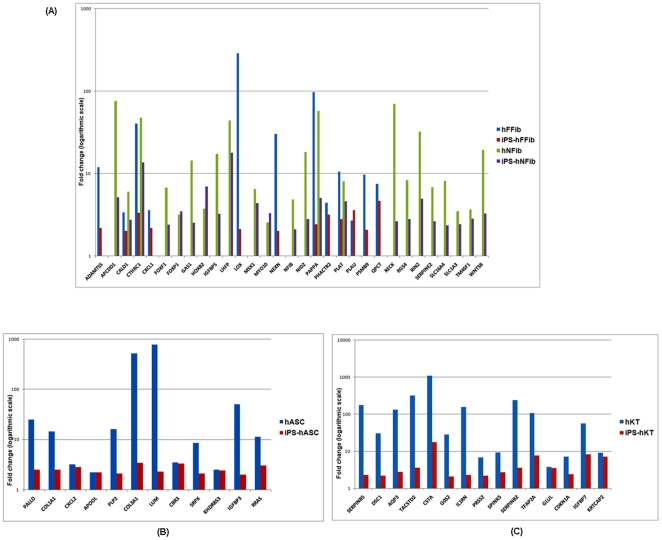
Residual signatures of the donor cell specific genes (upregulated in both hiPSCs and donor cells compared to hESC) in hiPSCs. (**A**) Expression fold-change of fibroblast specific genes in iPS-hFFib, hFFib, iPS-hNFib, and hNFib. (**B**) Expression fold-change of adipose cell specific genes in iPS-hASC and hASC. (**C**) Expression fold-change of keratinocyte specific genes in iPS-hKT and hKT.

Unsupervised hierarchical clustering of upregulated genes in hiPSCs and donor cells with respect to hESCs further confirmed the proximity of hiPSCs to their corresponding cell of origin (**Figure 5**) as compared to other donor cell types. For each set of iPS-donor cell types, IPA analysis was performed for functional annotation of the set of upregulated genes (**Supplementary Table S1-A** to **S1-D**). We clarified the role of these genes in various basic processes (cellular growth and proliferation, tissue development, cellular function, lipid metabolism, connective tissue development, DNA repair, cellular maintenance, etc). Next, we examined the expression of fibroblast [32], fat [33], [34], [35], [36], and keratinocyte [37] specific genes within the upregulated gene sets. We found significant residual gene expression of fibroblast (**Figure 6A**), adipocyte (**Figure 6B**), and keratinocyte genes (**Figure 6C**) within their corresponding hiPSCs. Specifically, fibroblast genes in **Figure 6A** such as PLAT and PLAU [32], [38] play important roles in remodeling the extracellular matrix and other functions in the coagulation system. Other fibroblast genes include CXCL1, which is involved in cell migration [32], and FOXF1 and FOXP1, which are forkhead family transcription factors expressed in fibroblasts [39]. CXCL2 in **Figure 6B**, also known as MIP-2 or macrophage inflammatory protein-2, PALLD, and COL1A1 are proteins expressed in adipocytes [34]. Among the keratinocyte-specific genes showed in **Figure 6C**, we found various keratins, transcription factors, and proteolytic enzymes (and their inhibitors) that are active in protein turnover and remodeling in keratinocytes, and which are not common to other cell types [37]. Taken together, our results demonstrate persistent donor cell gene expression within hiPSCs, and suggest a failure of reprogramming to efficiently silence the expression of these somatic genes.

### Downregulated Genes in hiPSCs and Donor Cells

**Figure 7:**
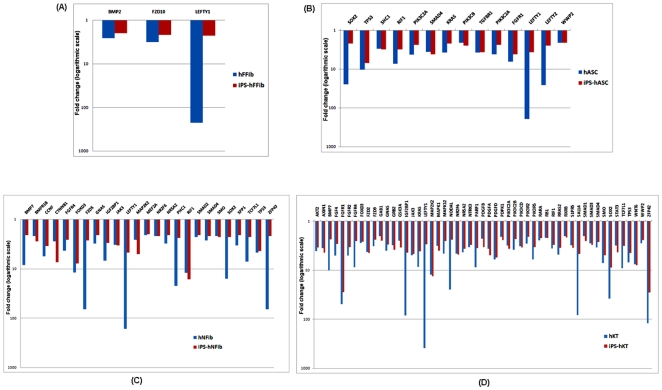
Residual signatures of the genes (downregulated in both hiPSCs and donor cells compared to hESC) involved in human embryonic stem cell pluripotency. (**A**) Expression fold-change in iPS-hFFib and hFFib. (**B**) Expression fold-change in iPS-hASC and hASC. (**C**) Expression fold-change in iPS-hNFib and hNFib. (**D**) Expression fold-change in iPS-hKT and hKT.

Ideally, the path to reprogramming should lead towards induction of embryonic genes that are responsible for maintaining an undifferentiated and highly proliferative state. To search for the embryonic genes that may be incompletely induced within hiPSCs, we analyzed the downregulated set of genes in both hiPSCs and their donor cells with respect to hESCs. IPA analysis was performed to functionally annotate these genes (**Supplementary Table S2-A** to **S2-D**). **Figure 7** shows the fold-change of selected genes that are involved in the hESC pluripotency. Overall, we observed incomplete induction of those genes needed to maintain an undifferentiated state in fibroblast-derived hiPSCs (**Figure 7A **and **7C**), fat-derived hiPSCs (**Figure 7B**), and keratinocyte-derived hiPSCs (**Figure 7D**). Specifically, LEFTY1 [40] is significantly downregulated in all the hiPSCs. SOX2 [41], RIF1, and TP53 [42] exhibited lower expression in all hiPSCs except iPS-hFFib. Another important embryonic marker gene, ZFP42, also known as REX1 [43], is downregulated in iPS-NFib and iPS-hKT.

### Genes with Opposite Expression in hiPSCs and Donor Cells

While comparing the gene expression profiles of the hiPSCs and donor cells *with respect to hESCs*, we came across a set of genes whose mode of regulation is *opposite* in hiPSCs as compared to their donor cells. A closer inspection of these genes, which are upregulated in hiPSCs but downregulated in donors and *vice versa*, reveals a gene expression pattern that is unique for the hiPSC state derived from different cell sources. **Supplementary Figures S1, S2, S3**, and **S4** show the expression pattern of these genes that uniquely define each hiPSC state. A detailed functional annotation of these set of genes is provided in **Supplementary Table S3**. These results suggest that hiPSCs also bear a unique pluripotent cell state as defined by their gene expression. A detailed inspection of these genes in the future will help characterize the hiPSCs, and better define the changes that occur during reprogramming.

## Discussion

In addition to their extraordinary potential for the field of regenerative medicine, hiPSCs provide a powerful system for studying the regulation of cell-fate transitions and the molecular programs that permit conversion of one cell type to another. This field has witnessed rapid growth in the development of safer and more efficient methods for deriving hiPSCs. However, in order to take advantage of the power of this new technology, it is important to more fully understand the character of these cells. We have executed a detailed investigation into the available transcriptional profiles of hiPSCs derived from fetal fibroblasts, neonatal fibroblasts, adipose cells, and keratinocytes. While the overall transcriptional profiles of hiPSCs share a common “signature” with hESCs, a subset of the gene profiles does suggest retention of “transcriptional memory” of the tissue of origin. Moreover, another subset of the gene expression pattern identifies the hiPSC state as unique from that of hESCs as well as from that of donor cells (Supplemental Figures S1, S2, S3, S4).

However it is possible that some of the results we highlight in this study may be attributed to varying culturing conditions, cell passage number, and viral vs. non-viral transfection techniques used across different laboratories. For example, iPS-hASC (viral), iPS-hFFib (non-viral), and iPS-hKT (viral) were cultured on irradiated mouse embryonic fibroblasts (MEFs), and Matrigel was used later for feeder free culture. In contrast, iPS-hNFib (non-viral) were cultured on gelatin for feeder-free conditions, while iPS-hKT were also cultured on human fibroblasts. Irrespective of these differences, it appears that *inherent* differences do indeed exist between hiPSCs and hESCs, and among hiPSCs themselves [14], [19], [20]. The variation in the techniques used in reprogramming will be an important consideration for future studies to assess whether they could significantly impact the overall findings of our current study. Another important area of concern for this study is the inherent heterogeneity in stem cell populations [44], [45]. Accordingly, incomplete reprogramming of hiPSCs, as demonstrated by this study, could reflect the fact that the cell population used in the microarray analysis contained contaminating subpopulations of cells that have not been completely reprogrammed, and thus give rise to expression signatures of the parental cells [46]. In other words, individual cells within a heterogeneous population are either reprogrammed or not, and contamination with the latter might be a cause of this “donor cell memory”. Contamination with large numbers of incompletely reprogrammed cells may explain the surprising gene expression results from keratinocyte-derived iPSCs, which were found to be significantly closer to their parental cells.

In this study, we sought to understand how similar the transcriptional profiles of hiPSCs are to their respective donor cells and to hESCs. Is there an epigenetic memory in hiPSCs that is related to their tissue of origin? Here, the word “epigenetic” is used according to Waddington's definition[47] in which he describes gene regulation and its consequence for developmental state, and “memory” refers to the residual gene activity patterns of the donor state within hiPSCs. Our analysis has clearly revealed the exact status of different hiPSC lines along the path of reprogramming: hiPSCs derived from fetal fibroblasts bear a reprogrammed status closest to hESCs, followed by adipose stem cells, neonatal fibroblasts, and keratinocyte-derived hiPSCs. Further, we also show that although most of the original epigenetic memory was erased in due course of reprogramming, there does exist some residual memory inherited from the donor cells which may affect the resulting hiPSCs, suggesting a deficit of reprogramming. This residual donor cell gene expression within hiPSCs may be a cause of the variations in teratoma formation thereafter [20]. It remains an interesting and pertinent question whether this epigenetic memory within hiPSCs induces them to differentiate into their original cell type more easily than into other somatic cell types.

To conclude, our data suggest that the reprogramming process does *not* de-differentiate the somatic cells completely to an ESC-state; further alteration or modification may therefore be necessary at the molecular level to reset the somatic nucleus *completely* to an embryonic state. This work has attempted to present a comprehensive analysis of available microarray data of hiPSCs, and has produced interesting observations that can be used as a guide for future reprogramming experiments. We hope that our results will also assist in the selection of optimal sources of donor cells for generating hiPSCs. Further, investigations to improve our understanding of the incompleteness in reprogramming will allow us to modify the methods for deriving hiPSCs best suited for clinical applications.

## Supporting Information

Figure S1 Unique set of genes (A) Upregulated in iPS-hFFib (red). (B) Downregulated genes in iPS-hFFib (green).
(0.06 MB TIF)

Figure S2 Unique set of genes (A) Upregulated in iPS-hASC. (B) Downregulated in iPS-hASC.
(0.07 MB TIF)

Figure S3 Unique set of genes (A) Upregulated in iPS-hNFib. (B) Downregulated in iPS-hNFib.
(0.07 MB TIF)

Figure S4 Unique set of genes (A) Upregulated in iPS-hKT. (B) Downregulated in iPS-hKT.
(0.07 MB TIF)

Table S1 Functional analysis of the upregulated genes in (A) iPS-hFFib and hFFib; (B) iPS-hASC and hASC; (C) iPS-hNFib and hNFib and (D) iPS-hKT and hKT
(0.18 MB XLS)

Table S2 Functional analysis of the downregulated genes in (A) iPS-hFFib and hFFib; (B) iPS-hASC and hASC; (C) iPS-hNFib and hNFib and (D) iPS-hKT and hKT
(0.17 MB XLS)

Table S3 Functional analysis of the (A1) upregulated genes in iPS-hFFib designating a unique hiPSC state; (A2) downregulated genes in iPS-hFFib designating a unique hiPSC state. (B1) upregulated genes in iPS-hASC designating a unique hiPSC state; (B2) downregulated genes in iPS-hASC designating a unique hiPSC state. (C1) upregulated genes in iPS-hNFib designating a unique hiPSC state; (C2) downregulated genes in iPS-hNFib designating a unique hiPSC state. (D1) upregulated genes in iPS-hKT designating a unique hiPSC state; (D2) downregulated genes in iPS-hKT designating a unique hiPSC state.
(0.12 MB XLS)

## References

[1] Stojkovic M, Lako M, Strachan T, Murdoch A (2004). Derivation, growth and applications of human embryonic stem cells.. Reproduction.

[2] Nishikawa S-i, Goldstein R, Nierras C (2008). The promise of human induced pluripotent stem cells for research and therapy.. Nature Reviews Molecular Cell Biology.

[3] Sipp D (2009). Gold standards in the diamond age: the commodification of pluripotency.. Cell Stem Cell.

[4] Swijnenburg R-J, Schrepfer S, Cao F, Pearl J, Xie X (2008). In vivo imaging of embryonic stem cells reveals patterns of survival and immune rejection following transplantation.. Stem Cells and Development.

[5] Swijnenburg R-J, Schrepfer S, Govaert J, Cao F, Ransohoff K (2008). Immunosuppressive therapy mitigates immunological rejection of human embryonic stem cell xenografts.. Proceedings of the National Academy of Sciences of the United States of America.

[6] Takahashi K, Yamanaka S (2006). Induction of pluripotent stem cells from mouse embryonic and adult fibroblast cultures by defined factors.. Cell.

[7] Lowry WE, Richter L, Yachechko R, Pyle AD, Tchieu J (2008). Generation of human induced pluripotent stem cells from dermal fibroblasts.. Proceedings of the National Academy of Sciences of the United States of America.

[8] Park I-H, Lerou P, Zhao R, Huo H, Daley G (2008). Generation of human-induced pluripotent stem cells.. Nature Protocols.

[9] Takahashi K, Tanabe K, Ohnuki M, Narita M, Ichisaka T (2007). Induction of pluripotent stem cells from adult human fibroblasts by defined factors.. Cell.

[10] Yu J, Vodyanik MA, Smuga-Otto K, Antosiewicz-Bourget J, Frane JL (2007). Induced pluripotent stem cell lines derived from human somatic cells.. Science.

[11] Aasen T, Raya A, Barrero M, Garreta E, Consiglio A (2008). Efficient and rapid generation of induced pluripotent stem cells from human keratinocytes.. Nature Biotechnology.

[12] Kim D, Kim C-H, Moon J-I, Chung Y-G, Chang M-Y (2009). Generation of human induced pluripotent stem cells by direct delivery of reprogramming proteins.. Cell Stem Cell.

[13] Loh YH, Agarwal S, Park IH, Urbach A, Huo H (2009). Generation of induced pluripotent stem cells from human blood.. Blood.

[14] Marchetto MCN, Yeo G, Kainohana O, Marsala M, Gage F (2009). Transcriptional signature and memory retention of human-induced pluripotent stem cells.. PloS ONE.

[15] Sun N, Panetta NJ, Gupta DM, Wilson KD, Lee A (2009). Feeder-free derivation of induced pluripotent stem cells from adult human adipose stem cells.. PNAS.

[16] Utikal J, Maherali N, Kulalert W, Hochedlinger K (2009). Sox2 is dispensable for the reprogramming of melanocytes and melanoma cells into induced pluripotent stem cells.. J Cell Sci.

[17] Yu J, Hu K, Smuga-Otto K, Tian S, Stewart R (2009). Human induced pluripotent stem cells free of vector and transgene sequences.. Science.

[18] Sun N LM, Wu J (2010). Human iPS cell-based therapy: considerations before clinical applications.. Cell Cycle (in press).

[19] Chin M, Mason M, Xie W, Volinia S, Singer M (2009). Induced pluripotent stem cells and embryonic stem cells are distinguished by gene expression signatures.. Cell Stem Cell.

[20] Miura K, Okada Y, Aoi T, Okada A, Takahashi K (2009). Variation in the safety of induced pluripotent stem cell lines.. Nature Biotechnology.

[21] Li J-Y, Christophersen N, Hall V, Soulet D, Brundin P (2008). Critical issues of clinical human embryonic stem cell therapy for brain repair.. Trends in Neurosciences.

[22] Yamanaka S (2009). A fresh look at iPS cells.. Cell.

[23] Maherali N, Hochedlinger K (2008). Guidelines and techniques for the generation of induced pluripotent stem cells.. Cell Stem Cell.

[24] Barrett T, Troup D, Wilhite S, Ledoux P, Rudnev D (2007). NCBI GEO: mining tens of millions of expression profiles–database and tools update.. Nucleic Acids Research.

[25] Gibbons F, Roth F (2002). Judging the quality of gene expression-based clustering methods using gene annotation.. Genome Research.

[26] Eisen MB, Spellman PT, Brown PO, Botstein D (1998). Cluster analysis and display of genome-wide expression patterns.. Proceedings of the National Academy of Sciences of the United States of America.

[27] Calvano S, Xiao W, Richards D, Felciano R, Baker H (2005). A network-based analysis of systemic inflammation in humans.. Nature.

[28] Gosiewska A, Yi CF, Brown LJ, Cullen B, Silcock D (2001). Differential expression and regulation of extracellular matrix-associated genes in fetal and neonatal fibroblasts.. Wound Repair and Regeneration.

[29] Zuk PA ZM, Ashjian P, De Ugarte DA, Huang JI (2002). Human adipose tissue is a source of multipotent stem cells.. Mol Biol Cell.

[30] Bunnell B, Flaat M, Gagliardi C, Patel B, Ripoll C (2008). Adipose-derived stem cells: isolation, expansion and differentiation.. Methods.

[31] Fuchs E (2007). Scratching the surface of skin development.. Nature.

[32] Rinn J, Bondre C, Gladstone H, Brown P, Chang H (2006). Anatomic demarcation by positional variation in fibroblast gene expression programs.. PLoS Genetics.

[33] Dahlman I, Linder K, Arvidsson Nordstrom E, Andersson I, Liden J (2005). Changes in adipose tissue gene expression with energy-restricted diets in obese women.. Am J Clin Nutr.

[34] Jernas M, Palming J, Sjoholm K, Jennische E, Svensson PA (2006). Separation of human adipocytes by size: hypertrophic fat cells display distinct gene expression.. FASEB J.

[35] Sjöholm K PJ, Olofsson LE, Gummesson A, Svensson PA (2005). A Microarray Search for Genes Predominantly Expressed in Human Omental Adipocytes: Adipose Tissue as a Major Production Site of Serum Amyloid A.. J Clin Endocrinol Metab.

[36] Urs S, Smith C, Campbell B, Saxton AM, Taylor J (2004). Gene expression profiling in human preadipocytes and adipocytes by microarray analysis.. J Nutr.

[37] Gazel A, Ramphal P, Rosdy M, De Wever B, Tornier C (2003). Transcriptional profiling of epidermal keratinocytes: comparison of genes expressed in skin, cultured keratinocytes, and reconstituted epidermis, using large DNA microarrays.. The Journal of Investigative Dermatology.

[38] Yih L-H, Peck K, Lee T-C (2002). Changes in gene expression profiles of human fibroblasts in response to sodium arsenite treatment.. Carcinogenesis.

[39] Chang H, Chi J-T, Dudoit S, Bondre C, van de Rijn M (2002). Diversity, topographic differentiation, and positional memory in human fibroblasts.. Proceedings of the National Academy of Sciences of the United States of America.

[40] Nakatake Y, Fukui N, Iwamatsu Y, Masui S, Takahashi K (2006). Klf4 cooperates with Oct3/4 and Sox2 to activate the Lefty1 core promoter in embryonic stem cells.. Mol Cell Biol.

[41] Fong H, Hohenstein KA, Donovan PJ (2008). Regulation of self-renewal and pluripotency by Sox2 in human embryonic stem cells.. Stem Cells.

[42] Miura T, Mattson MP, Rao MS (2004). Cellular lifespan and senescence signaling in embryonic stem cells.. Aging Cell.

[43] Mongan NP, Martin KM, Gudas LJ (2006). The putative human stem cell marker, Rex-1 (Zfp42): structural classification and expression in normal human epithelial and carcinoma cell cultures.. Mol Carcinog.

[44] Graf T, Stadtfeld M (2008). Heterogeneity of embryonic and adult stem cells.. Cell Stem Cell.

[45] Huang S (2009). Reprogramming cell fates: reconciling rarity with robustness.. BioEssays.

[46] Chang H, Oh P, Ingber D, Huang S (2006). Multistable and multistep dynamics in neutrophil differentiation.. BMC Cell Biology.

[47] Waddington CH (1957). The Strategy of the Genes; a Discussion of Some Aspects of Theoretical Biology (London: Allen & Unwin).

