# Supplementary figures and images for: Persistent Donor Cell Gene Expression among Human Induced Pluripotent Stem Cells Contributes to Differences with Human Embryonic Stem Cells
Source: PLoS One. 2010 Feb 1;5(2):e8975. doi: 10.1371/journal.pone.0008975 (PMC2813859; doi:10.1371/journal.pone.0008975)

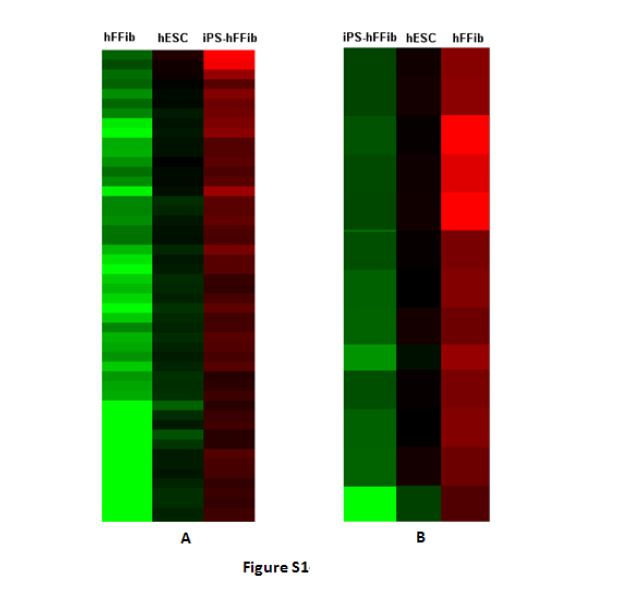

Supplement: Figure S1 — Unique set of genes (A) Upregulated in iPS-hFFib (red). (B) Downregulated genes in iPS-hFFib (green). (0.06 MB TIF) [file pone.0008975.s001.tif]

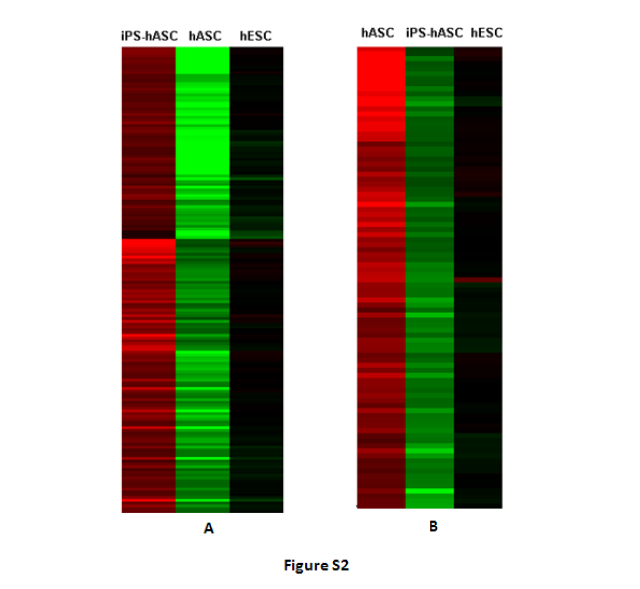

Supplement: Figure S2 — Unique set of genes (A) Upregulated in iPS-hASC. (B) Downregulated in iPS-hASC. (0.07 MB TIF) [file pone.0008975.s002.tif]

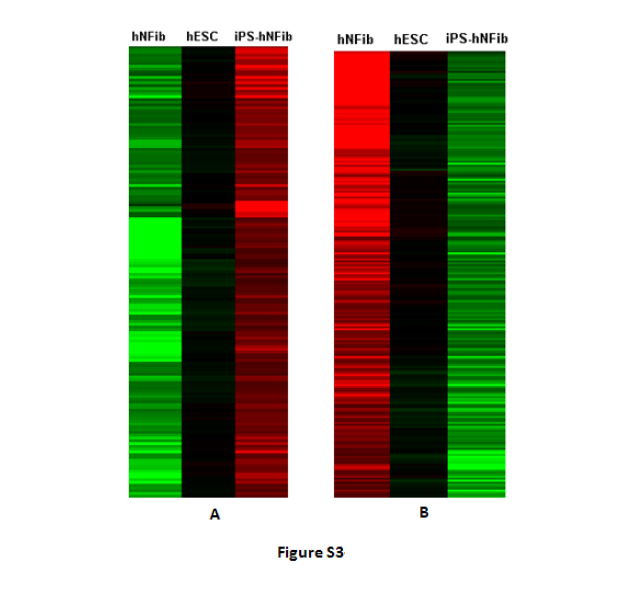

Supplement: Figure S3 — Unique set of genes (A) Upregulated in iPS-hNFib. (B) Downregulated in iPS-hNFib. (0.07 MB TIF) [file pone.0008975.s003.tif]

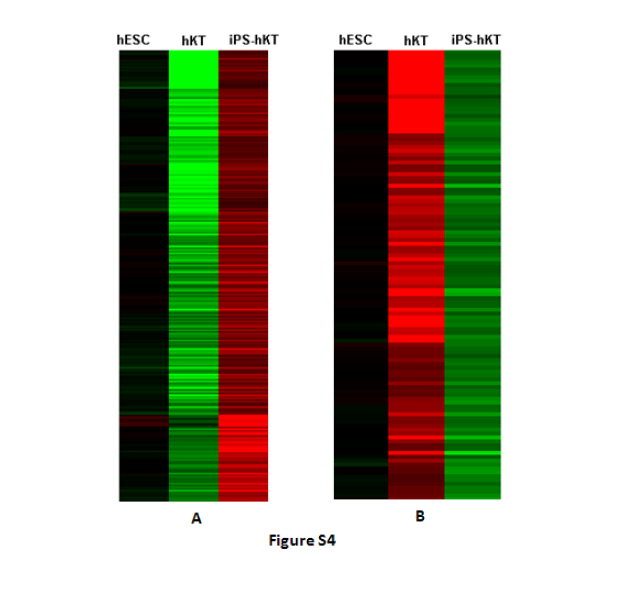

Supplement: Figure S4 — Unique set of genes (A) Upregulated in iPS-hKT. (B) Downregulated in iPS-hKT. (0.07 MB TIF) [file pone.0008975.s004.tif]
